# The absence of piriformis muscle, combined muscular fusion, and neurovascular variation in the gluteal region

**DOI:** 10.4322/acr.2020.239

**Published:** 2021-02-17

**Authors:** Matheus Coelho Leal, João Gabriel Alexander, Eduardo Henrique Beber, Josemberg da Silva Baptista

**Affiliations:** 1 Universidade Federal do Espirito Santo (Ufes), Departamento de Morfologia, Laboratório de Estudo em Morfologia Aplicada (LEMA), Vitória, ES, Brasil

**Keywords:** Anatomic Variation, Anatomy, Buttocks, Muscle, Piriformis Muscle Syndrome

## Abstract

The gluteal region contains important neurovascular and muscular structures with diverse clinical and surgical implications. This paper aims to describe and discuss the clinical importance of a unique variation involving not only the piriformis, gluteus medius, gluteus minimus, obturator internus, and superior gemellus muscles, but also the superior gluteal neurovascular bundle, and sciatic nerve. A routine dissection of a right hemipelvis and its gluteal region of a male cadaver fixed in 10% formalin was performed. During dissection, it was observed a rare presentation of the absence of the piriformis muscle, associated with a tendon fusion between gluteus and obturator internus, and a fusion between gluteus minimus and superior gemellus muscles, along with an unusual topography with the sciatic nerve, which passed through these group of fused muscles. This rare variation stands out with clinical manifestations that are not fully established. Knowing this anatomy is essential to avoid surgical iatrogeny.

## INTRODUCTION

The gluteal region contains important neurovascular and muscular structures that may impose diverse clinical and surgical approaches. Many studies have described and classified the anatomical variations in this region regarding the piriformis muscle (PM), sciatic nerve (SN), gluteus maximus (Gmax), medius (Gmed) and minimus (Gmin) muscles, and the regional neurovasculature. The prevalence of PM and SN variation is 16.9% in cadavers and 16.2% in surgical cases.^1^ The main reported findings are related to muscle fusions and the topography of the SN, which lead to clinical and surgical debate, especially on the piriformis syndrome (PS).^2^^-^^4^.

Over the years, these variations have been classified and distributed into different groups. Beaton and Anson^2^, in 1937, classically described the relationship between PM and SN into 6 types. More recently, Windisch et al.^3^ developed two ways to classify the PM presentation: three types depending on its morphology, and in four types depending on its fusion and different insertion areas.

In a routine dissection, we have found an unusual topography of the gluteal region with combined variations. In this paper, we aim to describe this unique variation and discuss the clinical implications, highlighting the need to recognize the possibility of such variations during diagnosis and therapeutic approach.

## CASE REPORT

The routine dissection of a right hemipelvis and its gluteal region of a male cadaver with approximately 35-50 years of age, fixed in 10% formalin, was performed at the Laboratorio de Estudos em Morfologia Aplicada (LEMA) of our institution. The body was donated by the Coroner’s Department of Espirito Santo State, according to the law 8.501/1992. The limb was previously disarticulated from the body and in the spine and the midline of the pelvis. The dissection was conducted from superficial to deep planes. Firstly, the skin and subcutaneous tissue were removed; the Gmax muscle was exposed. Secondly, a perpendicular section of the Gmax muscle fibers was made to expose the neurovascular structures and deep muscles. At the end of that procedure, we faced an unusual topography of the region. Thus, meticulous dissection was carried out to study the region with photographic documentation. To determine the anatomy variation, an additional perpendicular section was performed in the Gmed fibers, the pelvic floor and organs were dissected, and registration was made to present as results.

## AUTOPSY FINDINGS

**Figure 1:**
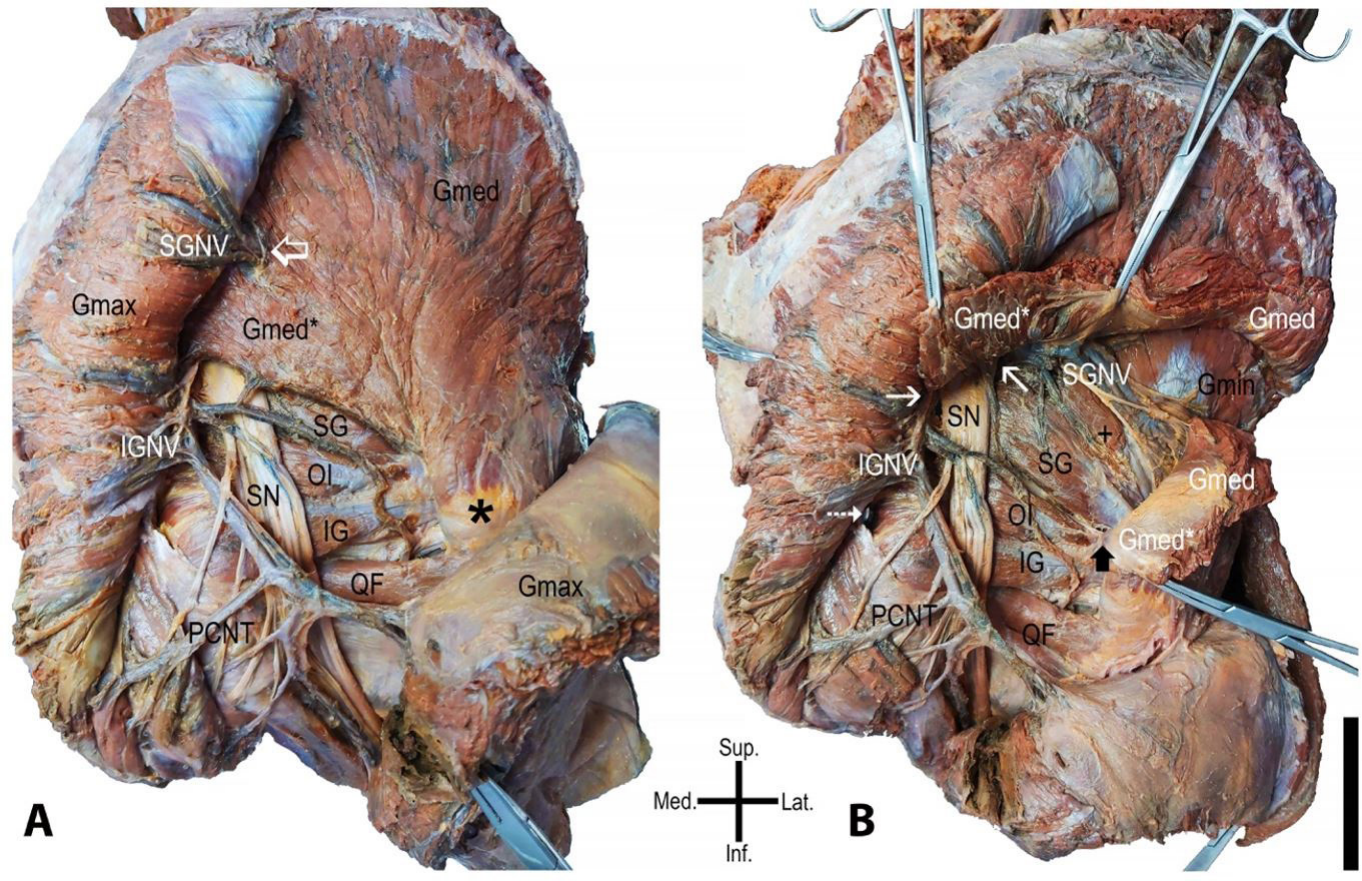
Photographs of the case. Gluteus maximus muscle is sectioned in **A**, and the gluteus medius muscle is sectioned in **B –** gluteus maximus muscle (Gmax); gluteus medius muscle (Gmed); extension part of the gluteus medius muscle (Gmed*); inferior gluteal neurovascular structures (IGNV); superior neurovascular gluteal structures (SGNV); superior gemellus muscle (SG); obturator internus muscle (OI); inferior gemellus muscle (IG); quadratus femoris muscle (QF); sciatica nerve (SN); posterior cutaneous nerve of the thigh (PCNT); gluteus minimum muscle (Gmin); fusion point between superior gemellus muscle and Gmin (+). The empty white arrow indicates the “ring-like” condensed connective tissue in the gluteus medius muscle. The asterisk refers to the greater trochanter. The thin white arrow indicates the greater sciatica foramen. The dotted white arrow points out the lesser sciatica foramen. The black arrow indicates the tendon fusion between Gmed* and obturator internus muscle. Scale bar 5cm.

The Gmax exhibited a usual aspect (Figure 1A). After his section and bending, we noticed the absence of a distinct PM. The Gmed was identified extending his attach inferiorly, in the lateral crest and margin of the sacrum beyond its usual extension. The superior gluteal vessels and nerve (SGVN) were seen passing through the Gmed and its extension to reach the Gmax. In this path, the SGVN seems to be fastened by a “ring-like” condensed connective tissue in the Gmed fascia (Figure 1B).

The SN and inferior gluteal vessels and nerve (IGVN) were identified after crossing the inferior margin of the Gmed extending part. Both the neurovascular structures were visualized running through the greater sciatica foramen and with regular distribution in the gluteal region. At this moment, the triceps coxae muscles and the quadratus femoris muscle shows usual characteristics (Figure 1B).

After sectioning the Gmed and its extension, we observed that his extended part attached with obturator internus muscle tendon (OI), no muscle was seen passing through the greater sciatic foramina, and the Gmin was fused with superior gemellus muscle (SG) (Figure 1B). The SN was found passing between the extending and overlapping parts of the Gmed and the muscle fusion between SG and Gmin, deep to the IGVN, and with the posterior cutaneous nerve of the thigh.

To ensure the PM variation, his proximal fixation was investigated in the inner face of the pelvis: pelvic floor fascia, organs, and neurovascular structures of the region were removed. The coccygeus muscle and the proximal insertion of the obturator internus muscles were visualized; however, no muscle insertion was found in the anterior surface of the sacrum.

In summary, we found an abnormal extension of the Gmed, an absence of a muscle passing through the greater sciatic foramen, a tendon fusion between the Gmed extended part and the OI, a muscle fusion between SG and Gmin, a variation in the neurovascular topography and the absence of a distinct PM. Thus, we decided to document the case with photographs and review the literature to discuss it.

## DISCUSSION

Considering the well-established studies describing the topography between PM and SN,^2^^,^^5^^-^^7^ and the modern ones describing detailed morphology of PM,^3^^,^^4^^,^^8^ the present case does not match their classification and description, which allows us to infer that it is unique. Although the extension part of the Gmed could be compared to what occurs in *Babirusas* (deer-pigs), which can observe a complete fusion between Gmed and PM,^9^ the origin of PM in the anterior surface of the sacrum is a well-established feature in hominoids,^10^ which lead us to define that it is a PM absence case.

Even though the absence of PM has been previously described - one case by Macalister^7^ and one case by Brenner et al.,^4^ with another doubtful reference of 2 cases in 6 specimens by Brenner et al.^4^, Duda et al.^11^, Nicholson et al.^12^ - in this case, when associating the absence of PM with the muscles fusions – we observe that such combination was not described in the literature. For example, Windisch et al.^3^ described the absence of PM associated with the absence of the inferior gemellus muscle, while SG was fused to OI.

Muscular fusions in the human gluteal region have been reported in the literature; Nicholson et al.^12^ documented fusions between OI and other tendons are common; Windisch et al.^3^ described as type 3 of their classification the merge between PM, Gmed, and OI. Regarding Gmin and SG, their fusion is also well established.^12^ Nevertheless, to the author’s knowledge, there is no description in the English language, a case that gathered the absence of PM, the tendon fusion between Gmed and OI, and the muscular fusion between SG and Gmin. On the functional behalf, those muscle fusion do not affect the hip range of motion in a health situation or an eventual muscle variation finding.^3^

Such variations can be justified through embryonic development. We believe that in our case, the close contact of the mesenchymal cells (ventral and dorsal) during the condensation (4th to 8th weeks of gestation) led to a union of their fibers or tendons.^3^

The anatomical variation in the topography of the PM and SN is among the causes of primary piriformis syndrome (PS). Although in the present specimen, we could not define the Gmed and Gmin extension as PM, the anomalous path of the SN through those structures had to be considered in cases where the patient has undetermined gluteal pain extended to the posterior part of the thigh.^3^^,^^13^

The ultrasound (US) and magnetic resonance imaging (MRI) are the main imaging diagnosis applied to the gluteal region, especially for invasive procedures. The US usually serves as a guide to invasive procedures in the deep gluteal region, like nerve blocks and posteriors surgical approaches.^4^ A routine MRI exhibits 12-20% of PM and SN variation, corroborating with the prevalence published in the meta-analysis by Smoll,^1^ and seems to be useful in optimal treatment planning.^13^^,^^14^

The knowledge of variations in the gluteal region has several clinical applications: hip trauma, PS, hip arthroplasty, buttocks reshaping, and anesthesia. In all of those, it needs to consider the possibility of overstretching the nerve in the entrapped areas causing or worsening symptoms, even leading to permanent nerve damage.^15^ Considering the high presence of variation in this region, clinicians and surgeons should be aware of the potential complications of medical or surgical interventions. Even though the prevalence of the piriformis variation in PS patients is not significantly different from the normal population - suggesting that this variation may not be as important in the pathogenesis of PS - a combined neurovascular and muscle variation, especially the presence of another PM tendon located inferiorly or deeply to the former, raises the need for attention in procedures in this area.^1^^,^^15^^,^^16^

## CONCLUSION

In conclusion, knowing the anatomy of the region and its variations is essential for a successful approach, good prognosis, and avoid possible iatrogeny. Thus, the unique variation presented in this article stands out, with clinical and surgical implications that were not fully established.
